# Douching With Water Works Device for Perceived Vaginal Odor With or Without Complaints of Discharge in Women With No Infectious Cause of Vaginitis: A Pilot Study

**DOI:** 10.1155/IDOG/2006/95618

**Published:** 2006-12-12

**Authors:** Ashwin J. Chatwani, Sarmina Hassan, Salma Rahimi, Stacey Jeronis, Vani Dandolu

**Affiliations:** ^1^Division of Gynecology, Department of Obstetrics and Gynecology, Temple University Hospital, 3401 N Broad Street, Philadelphia, PA 19140, USA; ^2^Department of Obstetrics and Gynecology, Temple University Hospital, 3401 N Broad Street, Philadelphia, PA 19140, USA; ^3^Division of Urogynecology and Pelvic Reconstructive Surgery, Department of Obstetrics and Gynecology, Temple University Hospital, 3401 N Broad Street, Philadelphia, PA 19140, USA

## Abstract

*Objective*. To determine if douching with Water Works device for 1 month can (1) lower or eliminate perceived vaginal odor by subject; (2) have any effects on vaginal ecosystem. *Methods*. Ten women with perceived vaginal odor with or without discharge, douched every day for 4 weeks in an open-label, nonrandomized pilot study. Primary outcome measures included perceived vaginal odor by subject, lactobacilli score from Nugent slide, and acceptance of the Water Works douching system. Secondary outcome included the safety of using this douching device. *Results*. At week 4, there was improvement in vaginal odor (*P* = .0006) and there was no significant change in lactobacilli score. *Conclusion*. Douching with Water Works device is associated with reduction or elimination of vaginal odor without adversely affecting the vaginal ecosystem.

## INTRODUCTION

Vaginal symptoms, including abnormal odor with or without complaints of discharge, are relatively common complaints for gynecologic consultation [1]. The evaluation of vaginal complaints is based primarily on the diagnosis of bacterial vaginosis (BV), trichomoniasis, and vaginal candidiasis (VVC) [2, 3]. Healthy women may experience vaginal discharge. The quantity of discharge appears to vary from woman to woman and during an individual woman's menstrual cycle. Normal vaginal fluid may have an odor that can be unpleasant [4]. Although BV is the most prevalent cause of vaginal discharge or malodor [5], studies in a variety of settings have demonstrated that neither BV nor any pathogenic microbe can be found in approximately a third of symptomatic women [6, 7]. The presence or absence of a microbe corresponds poorly with the presence or absence of symptoms. Clinicians are often faced with symptoms for which there are no obvious cause.

Douching has been reported with a variety of adverse outcomes [8–13]. Studies suggest that the effects of douching are modulated by the products used for douching [10, 11, 13], the reason, timing in relation to sexual activity and menstrual cycle [10, 11, 14]. A variety of commercial and homemade products are used for different reasons [15, 16]. The determinants of douching behavior are poorly understood even though it is widely practiced by many women in the United States [14, 17]. Although the question to douche or not to douche is still being debated [18, 19], possible benefits of douching are also suggested [13, 20].

Anecdotal evidence suggests that stainless steel has an effect in reducing odors and is so used to reduce odors on hands by chefs. Water Works is a medical grade, light weight (1 oz) stainless steel douching device that was developed to aid in the treatment of vaginal infections. A pilot clinical trial of subjects with BV using a stainless steel device showed potential for reducing and even eliminating vaginal odor. There is no known treatment for vaginal odor of nonbacterial origin. In addition pharmaceutical interventions are not appropriate in the face of the inability to identify a cause of odor. Purpose of our study was to evaluate whether douching with Water Works device is potentially beneficial for the treatment of abnormal vaginal odor while maintaining a normal vaginal ecosystem.

## MATERIALS AND METHODS

This open-label, nonrandomized pilot study was conducted at the Gynecology Clinic of Temple University Hospital. The study protocol was approved by the Temple University Institutional Review Board. Females attending the clinic who complained of abnormal vaginal odor were screened for vaginal infection. If clinical examination did not reveal any infection, the subject was invited to participate in the study. Major inclusion criteria included: (1) age 18 years and older; (2) complaint of abnormal odor with or without complaints of discharge, (3) odor scale 3 or greater (on a scale of 0–5, five being the worst); (4) subject not treated for BV, VVC with intravaginal, oral antifungal medications or antibiotics, within the last 14 days of enrollment. Exclusion criteria included: (1) pregnancy; (2) under treatment for cervical neoplasia; (3) abnormal anatomy or pathology of the vagina; (4) known HIV positive; (5) currently menstruating; (6) external factor (s) producing the odor and (6) body mass index (BMI) of 33 or greater.

## STUDY DESIGN

Women who met eligibility criteria were asked to participate in the study. Informed consent was obtained. All subjects were treated for a total of 4 weeks. Each patient underwent entry visit, 2nd visit after two weeks of douching and 3rd visit after 4 weeks of douching.

## CLINICAL METHODS DURING ENTRY VISIT

Standardized history and information on present genital symptoms, pregnancy history, contraceptive status and prior vaginitis history was taken. Same physician examined each patient and results were recorded on a standard form. Patient perceived vaginal odor scores were recorded. The vaginal pH was measured and vaginal swabs were collected from the posterior and lateral vaginal fornices in all patients. Saline wet mount and 10% KOH microscopy of vaginal secretions as well as Amine (“whiff”) test were performed. Papanicolaou (Pap) smear were done when results were not available or collected within 12 months. Gram stains for Nugent score slides were sent to a central laboratory to maintain the uniformity of the readings. Pregnancy test (urine HCG), OSOM Trichomonas rapid test or *Trichomonas vaginalis* culture (in subjects with a negative wet mount for trichomonads), *Chlamydia trachomatis* DNA test, *Neisseria gonorrhoeae* DNA test were done. Herpes simplex virus culture was done only if suspected. Vaginal yeast culture was done on all subjects.

## INVESTIGATIONAL PRODUCT

Water Works device is made of medical grade stainless steel. The kit includes a customized water container and tubing. It is a gravity-fed douching device, listed with the FDA that is Class I (21 CFR 884.5900). 32 ounces tepid tap water was used as douching fluid. Water container was hung approximately 3 feet above the vagina (eye level). It was designed to give a water pressure around 15 mm of mercury. Total douching time was approximately 2 min with constant manipulation of the douching device to make contact with all of the vaginal walls.

## TREATMENT REGIMEN

All subjects were instructed on how to use the treatment device and asked to douche once daily with the exception of the days of evaluation and menstruation. They were refrained from the use of other douches and intravaginal products (eg, feminine deodorant sprays, spermicides, tampons, and diaphragms) during the 4-week study period. They were not allowed to take oral/intravaginal antibiotics or antifungal agents during the 4-week study periods. Each subject kept a brief diary where they recorded their own assessment of vaginal odor and other vaginal symptoms during the whole length of study period. During 2nd and 3rd visits the speculum examination of the vagina were repeated. Swabs for vaginal pH, whiff test, saline and 10% KOH microscopy, were taken. Gram stain for Nugent score was sent to the central laboratory. Perceived vaginal odor score by the subjects were recorded.

## SAMPLE SIZE AND STATISTICAL ANALYSIS

This is a pilot study designed to estimate potential effect sizes so we can more reliably estimate sample size requirements for a randomized, controlled trial. Based upon data from 10 subjects, simple descriptive statistics were calculated. We used Student's *t*-test for continuous, paired variables.

## RESULTS

We screened 50 symptomatic women who were examined by the same gynecologist at Temple University Hospital, Department of OB/GYN between May 2005 and September 2005. Ten subjects met criteria and were enrolled for this pilot study. All the enrolled patients were African American. The mean age of the participants was 37.3 ± 10.15 (mean ± standard deviation) years.

**Figure 1:**
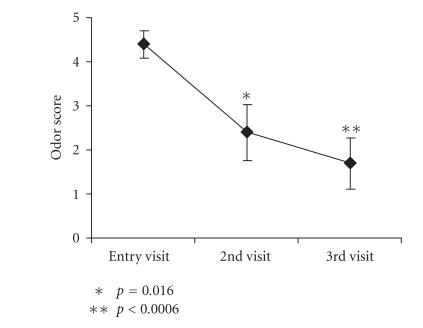
Perceived vaginal odor scored from the visual analog scale by subjects shows significant reduction (*P = .016) on 2nd visit with further reduction (**P < .0006) on 3rd visit. Data presented as mean ± standard error, *n* = 10.

**Figure 2:**
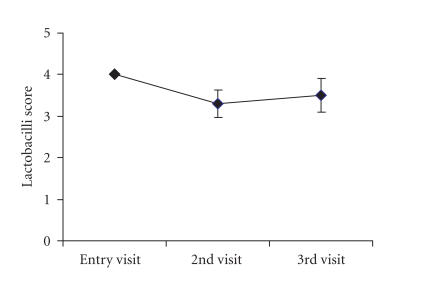
Lactobacilli score from the Nugent slide at entry, 2nd and 4th visit showed no significant (*P* > .05) decrease from baseline during 2nd and 3rd visit. Data expressed as mean ± standard error, *n* = 10.

At the screening visit (Entry visit), all women complained of strong vaginal odor. The mean vaginal odor score was 4.4± 0.3 (range 0–5). This odor was significantly reduced (*P* = .016) after 2 weeks of vaginal douching to 2.4 ± 0.6 (mean ± standard error). Odor score was further reduced to 1.7 ± 0.6 (*P* = .0006) after 4 weeks of douching (Figure 1). Five out of 10 subjects perceived complete abatement of odor at the end of study. Lactobacilli score from the Nugent slide was 4.0 ± 0 (mean ± standard error) on entry visit, and showed no significant decrease during 2nd (3.3 ± 0.3) and 3rd (3.5 ± 0.4) visits (Figure 2). Mean vaginal pH was 4 at both entry visit and final visit. At the end of treatment no statistically significant change of vaginal pH was noted.

Acceptability of the douching by the subjects was very high; one developed asymptomatic BV detected by Nugent score. All the subjects completed the study.

## DISCUSSION

The results of the present study, performed in 10 subjects with perceived vaginal odor with no infectious cause of vaginitis, confirm the efficacy and safety of the douching once daily for 4 weeks. At the first follow-up visit after 2 weeks of vaginal douching the perceived vaginal odor was 40% reduced. The second follow-up visit after 4 weeks of treatment further confirmed the effectiveness of douching by reducing the odor by 59% of baseline value. Half of the subjects showed complete recovery of their complaint of vaginal odor. Lactobacilli are the predominant bacteria in normal vaginal flora [21]. They maintain an acidic environment (pH of 4.5 or less in normal vagina), considered to be one of the protective mechanisms of the vagina [22]. Several in vitro and in vivo experiments in humans have examined how douching affects vaginal pH and microflora [18, 23]. Douching may wash away lactobacilli and protective factors and weaken the defense system in the vaginal ecology. In our study, douching induced no negative modification of the vaginal flora, as observed by lactobacilli score from Nugent slides and vaginal pH determination. Although douching has not been recommended for frequent use, previous study of daily douching caused no significant alterations in vaginal pH, which is in support of our finding [24]. BV is likely to occur when the balance between protective organism and potential pathogens is adversely altered.

Intensity and methods of douching, especially douching with pressure, have been associated with adverse outcomes. Therefore, the risk of ascending infection from the pressure of douching may be greatest around the time of ovulation when the cervical os is gaping and the mucus is thin. Recent study did not support the relation between douching and either gonococcal/chlamydial genital infection or pelvic inflammatory diseases [25]. The Water Works douching device is designed so that the water is directed downward away from the cervical os and also it is gravity fed to avoid the ascending infection. We did not observe any symptoms of pelvic infection during the length of study.

There are several limitations to our study. Vaginal discharge and odor were self-reported by participants, and at present, there are no validated instruments for objective assessment of vaginal symptoms. The size of small study population is our other limitation, but examination of all patients by the same physician and reading of slides for Nugent score by the same person at a central laboratory are the strengths of our study. Because this was a nonblinded study, there is the potential of reporting bias by both the clinician and the subject. Finally we did not attempt to follow up the patients to determine long-term effect of frequent douching.

Our findings suggest that further study of douching in a randomized, controlled trial is warranted. Future studies should also optimize the length and frequency of treatment. Nonetheless, our data suggest that the Water Works Douching Device is beneficial for the treatment of vaginal odor with no objective findings of vaginal infection while maintaining a normal vaginal ecosystem.
